# Catalyst poisoning influences from various functional groups of energy carriers towards electrochemical oxidation reactions on non-noble high-entropy alloy anodes in acidic media

**DOI:** 10.1080/14686996.2026.2653417

**Published:** 2026-04-24

**Authors:** Rafat Tahawy, Salma Aridha Muflihah, Kosuke Hara, Tatsuhiko Ohto, Hisanori Tanimoto, Tianshu Li, Mahmoud Abdelnabi, Samuel Jeong, Tomohiko Nishiuchi, Hajime Kimizuka, Akfiny Hasdi Aimon, Yoshikazu Ito

**Affiliations:** a Department of Applied Physics, Institute of Pure and Applied Sciences, University of Tsukuba, Tsukuba, Japan; b Central Metallurgical Research and Development Institute (CMRDI), Helwan, Egypt; c Department of Physics, Faculty of Mathematics and Natural Sciences, Institut Teknologi Bandung, Bandung, Indonesia; d Graduate School of Engineering, Nagoya University, Nagoya, Japan; e Physics Department, Faculty of Science, Ain Shams University, Cairo, Egypt; f Department of Chemistry, Graduate School of Science, The University of Osaka, Toyonaka, Japan; g Collaboration Research Center for Advanced Energy Materials, National Research and Innovation Agency, Institut Teknologi Bandung, Bandung, Indonesia; h Tsukuba Institute for Advanced Research (TIAR), University of Tsukuba, Tsukuba, Japan

**Keywords:** Non-noble metal, high entropy alloy, oxygen evolution reaction, oxidation reaction, anode, catalyst poisoning

## Abstract

Electrolytic synthesis of energy carriers using renewable energy and fuel cells that use energy carriers for regeneration are important technologies for achieving our carbon-neutral society. However, electrochemical reactions in electrolyte containing organic molecules significantly degrade the electrodes by catalyst poisoning (i.e. polymerization of organic molecules on the electrode surface). Such a situation can easily occur through the crossover phenomenon between the anode and cathode chambers. Thus, the understanding of the electrochemical reactivity of organic molecules on electrodes becomes important for utilizing energy carriers. This study investigated the electrochemical reactivity of various organic molecules with typical functional groups to understand the reaction mechanism and the subsequent catalyst poisoning in acidic media. While −OH groups on organic molecules do not cause significant degradations on a non-noble metal high-entropy alloy anode, the coexistence of −NH_2_ and C=O groups on organic molecules significantly degrades the anode because the generated polymers block the catalytically active sites. This knowledge will contribute to the effective design of catalysts/electrodes and benefit the communities in electrolytic synthesis and fuel cells.

## IMPACT STATEMENT

This study investigated the electrochemical reactivity of various energy carriers and catalyst poisoning in acidic media and also revealed the reaction mechanisms for electrolytic synthesis and fuel cells using energy carriers.

## GRAPHICAL ABSTRACT

**Figure:**
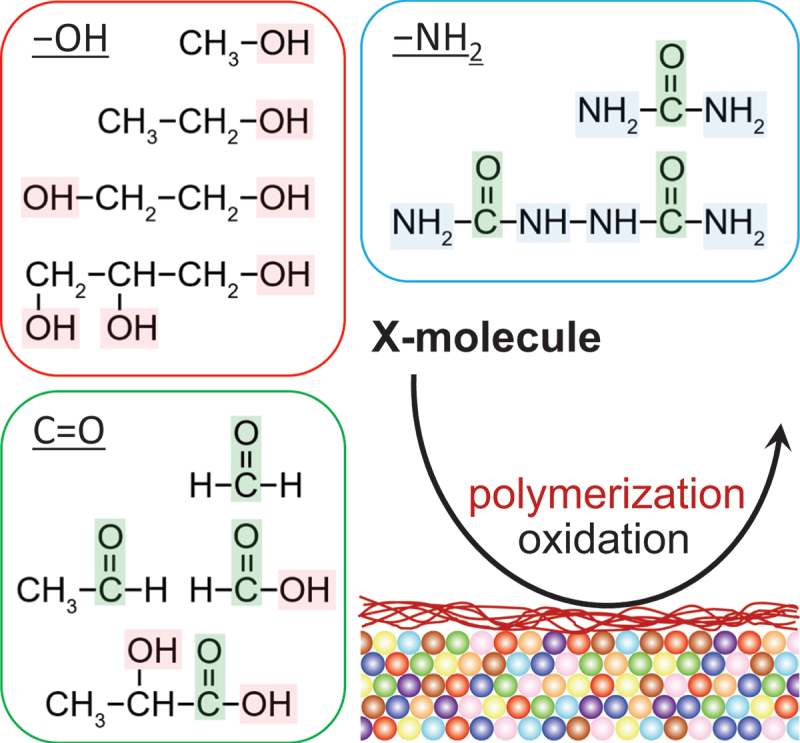

## 1. Introduction

Development of green hydrogen technology and fuel cell technologies relevant to energy carriers is a key to utilizing a variety of energy sources in a sustainable, carbon-neutral society [1–10]. Realizing such technologies, diverse energy carriers synthesized by electrolytic synthesis with renewable energy power and several regeneration methods using the energy carriers on fuel cells are necessary. However, impurities such as organic molecules or metal ions in electrolyte often cause severe degradation of the electrodes in electrolysis and fuel cell systems during operation [11,12], which rapidly decreases the electrodes’ lifetime [13]. Indeed, the development of an international hydrogen supply chain from hydrogen production areas with excess renewable energy to hydrogen consumption areas with limited renewable energy requires hydrogen transportation technology using energy carriers such as hydrogen-storing materials (i.e. organic chemical hydrides [14–19]). However, the organic chemical hydrides produced at the cathode chamber can cross over from the cathode chamber to the anode chamber through a proton exchange membrane, which causes severe catalyst poisoning on the anodes [16,20–22]. Additionally, electrodes in direct methanol fuel cells and other potential fuel cells using chemical fuels containing alcohol (ethanol), aldehyde (formaldehyde), carboxyl (formic acid), and amino (urea) groups are degraded by the crossover of organic molecules from the anode chamber to the cathode chamber [23,24]. Therefore, the reactivity of the functional groups of energy carriers with the catalyst must be investigated to reveal the reaction/resistance mechanism toward catalyst poisoning in acidic electrolyte containing simple energy carriers in comparison to the reaction/resistance mechanism using larger organic molecules with several functional groups such as alcohol (glycerol, ethylene glycol), aldehyde (acetaldehyde), carboxyl (lactic acid), and amino (biurea) groups. This will lead to the development of durable electrodes for use in electrolytic synthesis and fuel cells relevant to energy carriers.

During electrochemical reactions, crossover organic molecules in electrolyte accompany catalyst poisoning, which could be a major reason for the electrode’s degradation and prevent the long-term operation of electrolytic synthesis and fuel cells. Catalyst poisoning occurs due to undesirable polymerizations on the surface of electrodes/catalysts, which eventually block the catalytically active sites by the generated polymers [12,16,20]. One feasible solution to prevent degradation caused by the crossover of organic molecules is to increase the thickness of the proton exchange membrane [25]. However, the crossover cannot be completely prevented due to the diffusion/permeation of organic molecules involved in associated water, pressure differences between anode and cathode chambers, electroosmosis, etc. Another solution is to explore replacements of noble metal catalysts such as Pt and IrO_2_ that do not undergo undesirable electrochemical reactions with target organic molecules in acidic environments. Conventional non-noble metal catalysts such as single metals and bimetallic alloys are not actively involved in such electrochemical reactions in comparison to the noble metal catalysts, but they are either easily dissolved or completely passivated in acidic media under applied potentials. Thus, a potential candidate can be a non-noble metal-based high-entropy alloy (HEA) electrode because it is known to demonstrate a good balance between catalytic activity and chemical stability during oxidation reactions (i.e. oxygen evolution reaction (OER)) in acidic media [26–38]. Additionally, some HEAs decelerate the progress of electrochemical reaction due to the weak adsorption energy of the target organic molecules onto the HEA surface [12]. One example is a nonary alloy consisting of Ti, Cr, Mn, Fe, Co, Ni, Zr, Nb, and Mo elements (denoted as 9eHEA). In other words, catalytically inert elements such as passivation elements (for example, Nb) weaken the interaction between the target organic molecules and the HEA surface compared to the HEA containing catalytically active elements (for example, Ni) and noble metal catalysts such as IrO_2_ [12]. To design non-noble metal-based anodes that are resistant to catalyst poisoning, the influences of organic molecules on catalyst poisoning should be investigated. In particular, the role of functional groups of organic molecules that primarily interact with catalyst surfaces in the adsorption process must be clarified.

This study systematically investigated the electrochemical reactivity and the catalyst poisoning of a non-noble metal-based HEA with the functional groups (for example, alcohol, aldehyde, carboxyl, and amino groups) of energy carriers in an aqueous 0.5 M H_2_SO_4_ electrolyte containing an X-molecule such as methanol, ethanol, ethylene glycol, glycerol, acetaldehyde, formaldehyde, formic acid, lactic acid, urea, and biurea. The alcohol, carboxyl and amino groups exhibited strong adsorption energies on the HEA surface, and the coexistence of carboxyl and amino groups degrades the anode, and the generated polymers block the catalytically active sites on the HEA surface. Furthermore, the degree of catalyst poisoning relevant to the decrease in current density could depend on the characteristics of the functional groups in organic molecules acting as polymer precursors.

## 2. Experimental

### 2.1. Materials and synthesis method

Button-shaped HEA ingots were produced by arc melting in a pure Ar atmosphere (99.9999%, 60 kPa) [12,39]. Each alloy ingot (total weight: 10 g) was synthesized from pure parent metals: Ti (99.9 wt%), Cr (99.99 wt%), Mn (99.99 wt%), Fe (99.95 wt%), Co (99.995 wt%), Ni (99.995 wt%), Zr (99.9 wt%), Nb (99.9 wt%), Mo (99.9 wt%), and Cu (99.9 wt%), sourced from Hirano Seizaemon Syouten Co., Ltd., Rare Metallic Co., Ltd., Japan Metal Service, Furuuchi Chemical Co., and Materials Research Corp. Before melting the parent metal ingots, a piece of Ti was melted as a typical oxygen trap. Equal molar quantities of nine metals were thoroughly melted, along with the extra addition of Mn (10% equimolar amount), known for its low boiling point and high vapor pressure that causes it to evaporate. Following the total dissolution of the parent metals, the processing and remelting of the ingot were carried out no fewer than six times to synthesize uniformly blended alloys (i.e. homogenization). Then, the resulting ingots were sliced into several sheets using a mechanical slicer, and the surface of sliced sheets was polished by sandpapers for characterizations and measurements. In addition, a sliced sheet was mechanically crushed to small particles to observe the elemental distribution at nanoscale.

### 2.2. Characterization

The morphology and microstructure of the synthesized samples were analyzed using scanning electron microscopy (SEM, JEOL JCM −7000 NeoScope), transmission electron microscopy (TEM, JEOL JEM−ARM200F), and energy-dispersive X-ray spectroscopy (EDS; SDD Type, detection surface area 30 mm^2^, solid angle 0.26 sr). X-ray diffraction (XRD) analysis was performed with a D2 PHASER (Cu Kα1 radiation; λ = 1.5406 Å, Bruker). The sample surfaces’ organic compounds were analyzed via Fourier-Transform Infrared Spectrometer (FT-IR) spectroscopy (ThermoFisher Nicolet iS50) within the 4000 − 500 cm^−1^ range, employing an attenuated total reflectance method. The surface chemical states were analyzed through X-ray photoelectron spectroscopy (XPS; Shimadzu AXIS Ultra DLD) conducted using an Al Kα radiation source and an X-ray monochromator. Before the FT-IR, XPS, and SEM-EDS measurements, the samples were washed with ethanol after conducting a chronoamperometry (CA) test for 1 h and then dried at room temperature. The samples were pasted on a conductive carbon tape for the SEM-EDS measurements and XPS analysis, and the crushed particles were placed on a Cu grid with a carbon support film for TEM measurements.

### 2.3. Electrochemical measurements

Electrochemical tests for the oxygen evolution reaction (OER) in aqueous 0.5 M H_2_SO_4_ (pH = 0.5, 95%, Wako) containing X-molecules (X-molecule = methanol (99.8%, Wako), ethanol (99.5%, Wako), urea (99%, Wako), formic acid (88%, Wako), formaldehyde (37%, KANTO Chemical com. Inc.), biurea (Tokyo Chemical Industry Co., LTD.), acetaldehyde (90%, Wako), lactic acid (85–92%, Wako), glycerol (99.5%, Nacalai tesque), ethylene glycol (99.5%, Wako) were conducted in a standard three-electrode electrochemical setup. All experiments were carried out with an electrochemical workstation (Biologic VSP-300) at 25°C under O_2_ bubbling (99.9%). The cell was set up with a sat. Ag/AgCl electrode as a reference electrode, a 9eHEA working electrode as an anode, and a graphite rod as a counter electrode. 0.5 M concentration of X-molecules was added into 0.5 M H_2_SO_4_ electrolyte. The potentials were referenced to the reversible hydrogen electrode (RHE) through the equation: $E\left(\mathrm{RHE}\right)=E\left(\mathrm{Ag}/\mathrm{AgCl}\right)+0.059\times \mathrm{pH}+0.197$. Prior to cyclic voltammetry (CV) measurements, the anodes underwent electrochemical activation through 10 CV cycles within the potential ranges of 0.0–2.2 V (vs. RHE) for HEAs at a scan rate of 5 mV s^−1^. Then, the CV was recorded at a scan rate of 10 mV s^−1^. The double-layer capacitance (C_dl_) was determined from the CV curves obtained at scan rates ranging from 40 to 190 mV s^−1^ in the non-Faradaic regions (0.1–0.3 V (vs. RHE) in acidic conditions. Chronoamperometry (CA) experiments were performed for a target current density of 100 mA/cm^2^ over 50 h or 1 h for FT-IR measurements. Electrochemical impedance spectroscopy (EIS) tests were conducted at 1.8 V (vs. RHE) with a frequency spectrum of 100 μHz to 1 MHz. Note that same 9eHEA anode (surface area: 0.438 cm^2^) for all CV, C_dl_, and EIS measurements was intentionally employed for fair comparisons, and the 9eHEA surface was polished before the measurements.

### 2.4. Density functional theory (DFT) calculations

DFT calculations were performed with the VASP code [40] using the projected augmented wave method [41] and Perdew-Burke-Ernzerhof as the exchange-correlation functional [42]. The plane-wave energy cutoff was set to 400 eV. As the atomic-level structure of 9eHEA is unknown, we adopted a high-throughput protocol with the aid of the machine learning force field (MLFF) using the Gaussian approximated potential [43] and smooth overlap of atomic potential [44] schemes as previously described [12]. The MLFF was constructed from 30 ps-long DFT−MD simulations at 3000 K for a cubic cell (edge length: 15 Å) containing 216 atoms (24 atoms per species). We ran 10 ns classical MD simulations for the same system with the constructed MLFF using the LAMMPS package [45] and selected seven snapshots. Subsequently, we further ran 5-ps DFT−MD simulations and optimized the bulk structures, including the unit cell vectors. Seven surface slab models were created from each bulk structure by inserting a vacuum at *z* = 0, 2, 4, 6, 8, 10, and 12 Å, respectively. We obtained 7 (bulk structures) × 7 (surfaces) × 2 (upsides and downsides of the slab model) = 98 surface models for 9eHEA. To prepare the oxidized surfaces, we added O atoms to all surface atoms of the slab model containing the active site (Ni) with the highest oxygen evolution reaction activity. We employed Grimme’s D3(BJ) scheme [46,47] for dispersion correction to accurately estimate the adsorption energies of glycerol and urea on the oxidized 9eHEA surface.

## 3. Results and discussion

### 3.1. Material characterizations

**Figure 1.**
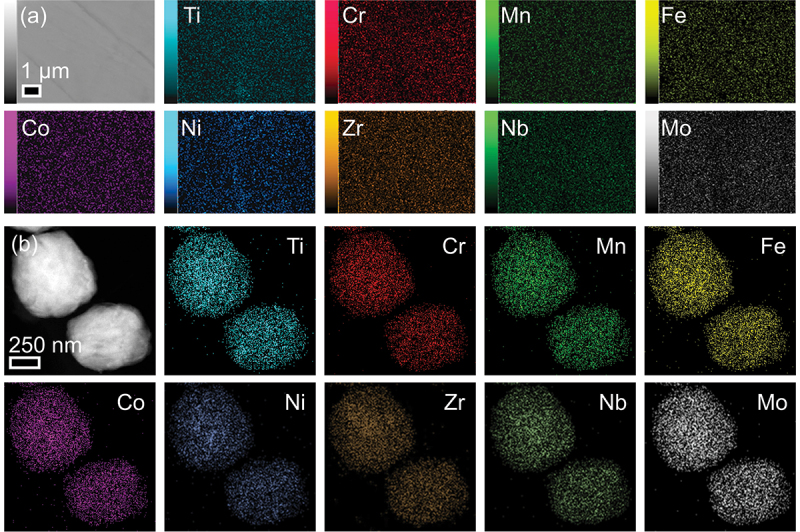
(a) SEM-EDS mapping and (b) STEM-EDS mapping of 9eHEA. Scale bars were 1 μm and 250 nm, respectively.

A 10 g button-shaped 9eHEA ingot was produced from the pure parent metals using a standard arc melting method. The ingots were sliced into sheets for use as electrodes (Figure S1). SEM-EDS measurements confirmed a smooth surface without distinct grain boundaries or elemental segregations (Figure 1a). Moreover, high-resolution TEM images of mechanically crushed 9eHEA particles showed crystal lattices [39], while an uniform elemental distribution at the nanoscale was also confirmed by a dark-field scanning transmission electron microscopy (DF-STEM) measurement (Figure 1b). XRD spectra of 9eHEA demonstrated the body-centered cubic (*bcc*)-like phase from the four-elemental alloys of Ti, Zr, Nb, and Mo; face-centered cubic (*fcc*) phase from the five-elemental alloys of Cr, Mn, Fe, Co, and Ni; as well as additional phases such as C14 and σ (Figure S2) [48,49]. These C14 and σ phases could be generated by introducing Ti, Zr, Nb, and Mo into CrMnFeCoNi alloys [50,51]. In addition, XPS measurement confirmed that the surface state of 9eHEA was metallic for all elements (Figure S3).

### 3.2. Electrochemical OER performances with X-molecules under acidic conditions

**Figure 2.**
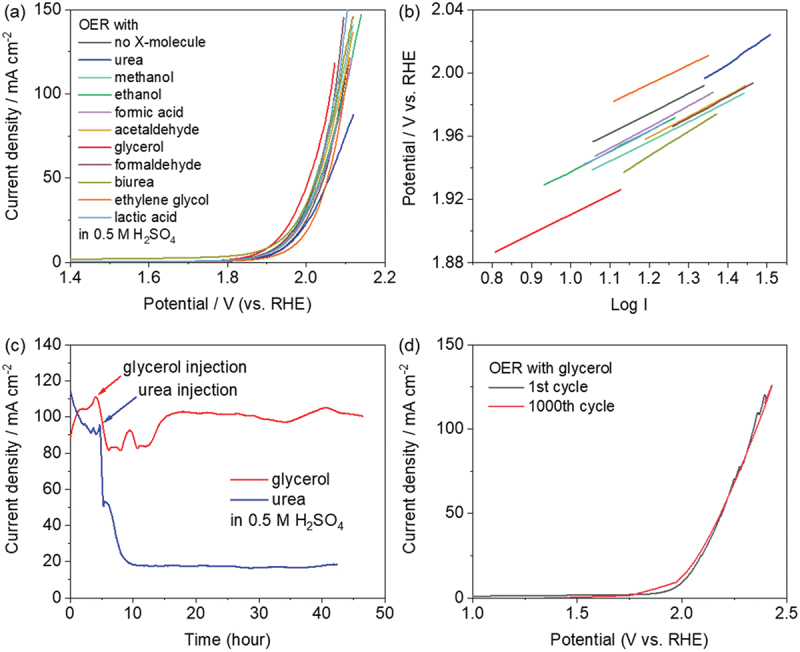
(a) CV measurements and (b) the Tafel slope of 9eHEA anode in aqueous 0.5 M H_2_SO_4_ electrolyte containing X-molecules or without X-molecule. (c) ca measurements of 9eHEA anode in aqueous 0.5 M H_2_SO_4_ electrolyte with the glycerol and urea injection. (d) Cycling stability test of 9eHEA anode in aqueous 0.5 M H_2_SO_4_ electrolyte containing glycerol.

Electrochemical OER performance of 9eHEA (with the same electrode and same surface, which was polished after each measurement) in an aqueous 0.5 M H_2_SO_4_ electrolyte, both with and without the X-molecules, was investigated with iR compensation (85%). Note that the oxidation of X-molecules in 0.5 H_2_SO_4_ electrolyte does not proceed well without heating, and 0.5 M of X-molecules was considered as the minimum fuel crossover concentration estimated from the full cell experiments [52] (see more details in supplementary discussion). Cyclic voltammograms were recorded after the CV activation, and the balanced performance between catalytic activity and chemical stability, unlike ordinary phenomena such as either dissolution or complete passivation on non-noble metal catalysts and bimetallic alloy catalysts, was observed. (Figure 2a and Table S1). 9eHEA in 0.5 M H_2_SO_4_ electrolyte with glycerol demonstrated the highest activity, whereas 9eHEA in 0.5 M H_2_SO_4_ electrolyte with urea demonstrated the lowest activity. The potentials at 100 mA/cm^2^ current density of 9eHEA in 0.5 M H_2_SO_4_ electrolyte with X-molecules (glycerol, formaldehyde, lactic acid, biurea, methanol acetaldehyde, acetaldehyde) were lower than those without the X-molecules, and the potentials at 100 mA/cm^2^ current density of 9eHEA in 0.5 M H_2_SO_4_ electrolyte with X-molecules (ethanol, formic acid, ethylene glycol, urea) were higher than those without the X-molecules. However, the differences in comparison to the performances without X-molecules were very small except for glycerol and urea (Figure 2a and Table S1). Indeed, the Tafel slopes of 9eHEA in 0.5 M H_2_SO_4_ electrolyte without (126 mV dec^−1^) and with X-molecules (120–134 mV dec^−1^) were quite similar except for urea and biurea (175 and 156 mV dec^−1^) (Figure 2b and Table S1). In addition, the *C*_dl_, which is almost proportional to the effective electrochemically active surface area (ECSA), was estimated in a non-Faradaic potential window (0.1−0.3 V vs. RHE) in 0.5 M H_2_SO_4_ electrolyte with (21–38 mF/cm^2^) and without X-molecules (22.0 mF/cm^2^) and thereby all *C*_dl_ values were quite similar (Figure S4 and Table S1). On the other hand, the electrochemical impedance measurements were conducted at 1.8 V (vs. RHE) to estimate the charge-transfer resistance (*R*_ct_) in 0.5 M H_2_SO_4_ electrolyte with and without X-molecules, and they demonstrated the large differences B). The *R*_ct_ values of 9eHEA in a 0.5 M H_2_SO_4_ electrolyte with X-molecules (450–780 ohm) estimated from an equivalent circuit consisting of a parallel combination with a *R*_ct_ and *C*_dl_ were lower than those without X-molecules (861 ohm). This indicates that oxidation reactions of X-molecules could occur. The *R*_ct_ value of 9eHEA in 0.5 M H_2_SO_4_ electrolyte with urea (928 ohm) was higher, which indicated that the oxidation reactions were impeded.

These results showed that ones with similar functional groups demonstrated similar performances, and the others with similar functional groups demonstrated different performances. X-molecules with alcohol groups (methanol, ethanol, ethylene glycol, and glycerol) have similar functional groups. Among these, glycerol, which has the largest number of −OH groups, demonstrated the highest performance. A higher performance with a large number of functional groups was also found in the X-molecules with amino and carboxyl groups (urea and biurea). On the other hand, X-molecules with aldehyde and carboxyl groups (formaldehyde, acetaldehyde, formic acid, and lactic acid) have similar functional groups but did not demonstrate significant performance differences. This could be attributed to the quick oxidation of aldehyde groups to carboxyl groups at the oxidation potentials, which is consistent with the electrochemical impedance measurements (Figure S5 and Table S1). Thus, the electrochemical reactivity depends on the characteristics of the functional groups in organic molecules.

The largest changes were observed on the test with glycerol and urea. Thus, the influence of these substances was further investigated to understand the reaction mechanism. The CA durability of the 9eHEA anodes in 0.5 M H_2_SO_4_ electrolyte at ~100 mA/cm^2^ current density with glycerol or urea injection (0.5 M concentrations) over 40 h was investigated (Figure 2c). The current density dropped after the glycerol injection, but the current density gradually recovered and was maintained for over 30 h. This durable behavior was also observed in 1000 cycling CV measurements in 0.5 M H_2_SO_4_ electrolyte containing glycerol without iR compensation (due to changes over time in the anode’s surface state and glycerol concentration in the electrolyte) (Figure 2d). Conversely, the current density dropped steeply after the urea injection, but the current density did not recover.

### 3.3. Investigation of catalyst poisoning by X-molecules under acidic conditions

**Figure 3.**
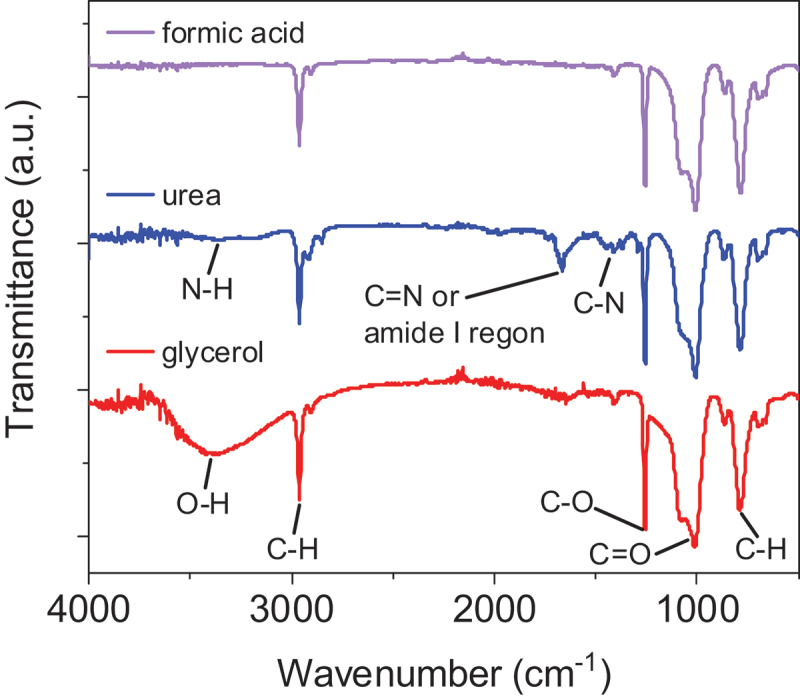
FT-IR results on the surface of 9eHEA anode tested in aqueous 0.5 M H_2_SO_4_ electrolyte containing formic acid, urea and glycerol from 1 h ca measurements.

To understand how functional groups affect the catalyst poisoning of 9eHEA, the polymers generated on the surface were investigated by FT-IR spectroscopy after the 1 h CA test in 0.5 M H_2_SO_4_ electrolyte containing the X-molecules. Aliphatic hydrocarbon polymer characteristics, such as −CH_2_ and −CH_3_ stretching vibrations at approximately 2850 and 2919 cm^−1^, as well as the oxidized functional groups (C−O, C=O) were detected on the HEA surface with all X-molecules. This evidence directly supports the formation of polymer (Figure 3 and Figures S6-S7). FT-IR data obtained from the test with X-molecules with alcohol groups (ethanol, ethylene glycol, glycerol) showed an −OH functional group, while FT-IR data obtained from the test with X-molecules with amino groups (urea and biurea) showed a −NH_2_ functional group. However, FT-IR data obtained from the test with X-molecules containing aldehyde and carboxyl groups simply showed the polymer features without −OH and −NH_2_ functional groups. Moreover, the surface state of 9eHEA anode after the CA test was investigated with XPS. The XPS detected the polymer characteristics (Figure S8). The 9eHEA anode tested with glycerol showed C–C and C−H bonds as well as −OH bonds, while the 9eHEA anode tested with urea showed C–C, C−H, C=O, and COO bonds as well as C−N and N−H bonds. These suggest that the initial functional groups in glycerol and urea were preserved in the polymer and some of −OH and C=O groups in glycerol and urea underwent oxidation. In addition, considering the weak XPS signals of underlaying 9 elements and uniform polymer formation on the surface confirmed by TEM [12], the polymer covers the entire 9eHEA surface.

The SEM-EDS analysis of 9eHEA after the 1 h CA test in 0.5 M H_2_SO_4_ electrolyte with X-molecules revealed large changes in surface morphology after the CA test compared to that before the CA test and without X-molecules (Figures S9-S19 and Table S2). These results suggest that the surface elements were involved in the oxidation reaction with organic molecules and then the surface elements were probably stripped off by X-molecules due to the increase of leaching amount in the existence of organic molecules (Figure S20), and the stripped atoms could work as polymerization initiators [12]. The polymerization occurred during the oxidation reaction, and the generated polymers gradually blocked the catalytically active sites on the HEA surface.

### 3.4. Understanding of adsorption mechanism using DFT calculations

**Figure 4.**
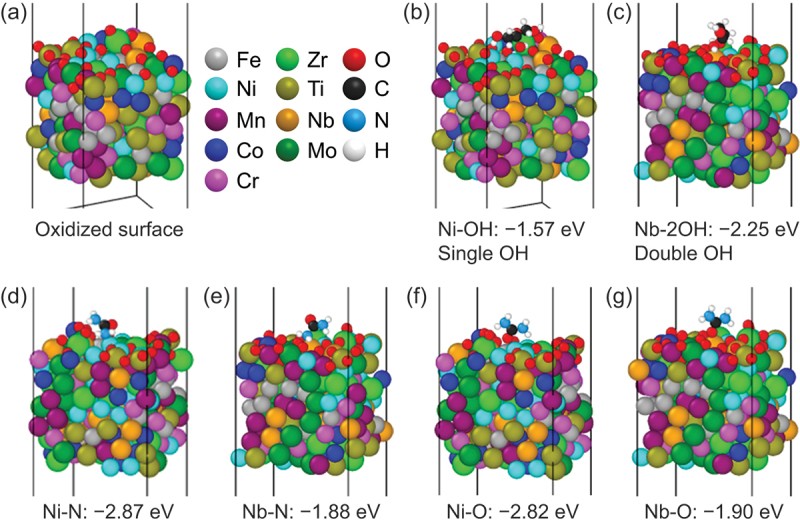
DFT calculations of glycerol and urea adsorption on an oxidized 9eHEA model. (a) Oxidized surface model with color codes of elements. (b) Single −OH adsorption on Ni, (c) double −OH adsorption on same Nb, (d) −NH_2_ adsorption on Ni, (e) −NH_2_ adsorption on Nb, (f) C=O adsorption on Ni, (g) C=O adsorption on Nb.

The adsorption energies of the glycerol and urea on the 9eHEA surface were investigated using DFT calculations to understand the subsequent polymer formation after the adsorption. In our previous report [12,39], the 9eHEA structures were optimized through high-throughput DFT calculations, and 2,677 sites on the 9eHEA surface were examined for OER. Accordingly, the most catalytically active sites and catalytically inactive sites were identified as Ni and Nb, respectively, whose active sites could appear on the interface between different phases and glass structure suggested by the analysis with Voronoi tessellation [53] (Figure S21). In addition, Nb was the most electrochemically stable passivation element with the highest atomic concentration (Nb: 29.1 at.%) at the 9eHEA surface at OER potential revealed by in situ XPS measurements [39]. Thus, Ni and Nb represent the 9eHEA characters and were selected for the target. To simulate the actual situation at oxidation conditions, the optimal surface structure was oxidized for further investigations (Figure 4a and Figure S22).

Single, double, and triple −OH groups on glycerol were adsorbed on the top of Ni and/or Nb on the surface in an upright direction with the adsorption site of the O from the −OH group (Figure 4b-c and Figure S23). In this position, the adsorption of a single −OH group of glycerol (from −1.77 to −1.57 eV) was weaker than the adsorption of double −OH groups of glycerol, and the adsorption of double −OH groups of glycerol on the Ni and Nb tops as bridge structures (−2.65 eV) was stronger than that of double −OH groups of glycerol on the same Nb top (−2.25 eV). Moreover, as a low-probability phenomenon, triple −OH groups of glycerol were also strongly adsorbed on the Ni and Nb as bridge structures (−2.11 eV) (Figure 4c and Figure S23). The −NH_2_ and C=O groups of urea were adsorbed on the Ni top or Nb top on the surface in an upright or recumbent direction with an adsorption site of N or O from CO(NH_2_)_2_ (Figure 4d-g and Figure S24-S25). In the upright and recumbent positions, the adsorption of the −NH_2_ group of urea on the Nb top (−1.88 eV and −1.64 eV) was weaker than that on the Ni top (−2.87 eV and −1.88 eV). The adsorption of the C=O group of urea was similar to that of −NH_2_ groups of urea on the Nb and Ni tops (from −2.82 to −1.83 eV). This indicates that urea can adsorb onto the surface in any position. Considering that the passivation element such as Nb significantly contributes to suppressing the adsorption of toluene (−CH_3_) (from −1.30 to −0.36 eV) and the subsequent polymerization on the 9eHEA surface [12], the adsorption ability of −OH, −NH_2_, and C=O groups on Nb was surely weakened by ~0.5 to 1 eV compared to Ni. However, Nb does not effectively work to prevent the adsorption and polymerization of glycerol and urea, and thus the −OH, −NH_2_, and C=O groups work as good adsorption sites. In other words, the adsorption of −OH, −NH_2_, and C=O groups is still strong and is considered as chemisorption [54]. Indeed, such strong adsorption characteristics rapidly decrease the current density of 9eHEA in electrolyte containing glycerol and, in particular, urea (absorption energy of −2.87 and −2.82 eV on Ni) strongly blocks the catalytically active Ni sites, further decreasing the current density in electrolyte containing urea compared to that in electrolyte containing glycerol (adsorption energy of −1.57 eV on Ni) (Figure 2c).

The difference between glycerol and urea was found in the recovery of current density (Figure 2c), and it could be attributed to the size of the molecules, the number of functional groups in the generated polymer, and the functional groups’ combinations. Since the dipole moments of glycerol and urea were nearly identical (3.42 ~ 4.42 Debye for glycerol, depending on the orientation of OH bonds, and 3.53 Debye for urea), the larger molecule (glycerol) experienced greater resistance to movement in 0.5 M H_2_SO_4_, while the smaller molecule (urea) could easily move to the anode surface. Additionally, the generated polymer from urea should have many functional groups, such as −NH_2_ and C=O groups, so that the generated polymers from urea adhere persistently to the surface anchored by these groups. Thus, the current density decreased and was not recovered (Figure 2c). Conversely, the adsorption rate of glycerol to the surface and desorption rate of the generated polymers from glycerol would balance, and thus the current density with glycerol was recovered (Figure 2c). This facilitation of desorption of glycerol/polymers is well supported by the DFT result that the adsorption strength of a single −OH group of glycerol (most probable adsorption manner) is much weaker than that of −NH_2_ and C=O groups of urea. Furthermore, since acetaldehyde, formaldehyde, formic acid, and lactic acid, which have a single C=O group with/without a −OH group(s), do not cause severe degradation on the CV and CA results (Figure 2a and Figure S6), the coexistence of −NH_2_ and C=O groups could enhance the degradation. Therefore, the adsorption energy of the functional groups and the interplay between the functional groups in the organic molecule and the elements on the surface play an important role in catalyst poisoning.

## 4. Conclusions

We systematically investigated how various functional groups influence electrochemical reactions and subsequent catalyst poisoning on a non-noble metal high-entropy alloy anode in an aqueous 0.5 M H_2_SO_4_ electrolyte containing methanol, ethanol, ethylene glycol, glycerol, acetaldehyde, formaldehyde, formic acid, lactic acid, urea, and biurea. We found that the −OH groups do not significantly degrade the anode performance, whereas the coexistence of −NH_2_ and C=O groups decreases the anode performance. These differences could be attributed to the coexistence of different functional groups and the nature of the generated polymer, which is affected by the target organic molecules acting as polymer precursors. These findings will contribute to electrolytic synthesis and fuel cells operating with energy carriers for the development of our carbon-neutral society.

## Supplementary Material

Supplemental Material
