# Supplementary material for: Catalyst poisoning influences from various functional groups of energy carriers towards electrochemical oxidation reactions on non-noble high-entropy alloy anodes in acidic media
Source: Sci Technol Adv Mater. 2026 Apr 24;27(1):2653417. doi: 10.1080/14686996.2026.2653417 (PMC13148096; doi:10.1080/14686996.2026.2653417)
Supplement: Supplemental Material [file TSTA_A_2653417_SM8360.docx]

**Supplementary material**

Catalyst poisoning influences from various functional groups of energy carriers towards electrochemical oxidation reactions on non-noble high entropy alloy anodes in acidic media

Rafat Tahawy^a,b^, Salma Aridha Muflihah^a,c,†^, Kosuke Hara^d,†^, Tatsuhiko Ohto^d,^*, Hisanori Tanimoto^a^, Tianshu Li^a^, Mahmoud Abdelnabi^a,e^, Samuel Jeong^a^, Tomohiko Nishiuchi^f^, Hajime Kimizuka^d^, Akfiny Hasdi Aimon^c,g^, Yoshikazu Ito^a,h,^*

^a^Department of Applied Physics, Institute of Pure and Applied Sciences, University of Tsukuba, 1-1-1 Tennodai, Tsukuba, Ibaraki, 305-8573, Japan

^b^Central Metallurgical Research and Development Institute (CMRDI), P.O. Box 87 Helwan, 11421, Egypt

^c^Department of Physics, Faculty of Mathematics and Natural Sciences, Institut Teknologi Bandung, Bandung, 40132, Indonesia

^d^Graduate School of Engineering, Nagoya University, Furo-cho, Chikusa-ku, Nagoya, Aichi 464-8603, Japan

^e^Physics Department, Faculty of Science, Ain Shams University, Cairo 11566, Egypt

^f^Department of Chemistry, Graduate School of Science, The University of Osaka, 1-1 Machikaneyama, Toyonaka, Osaka 560-0043, Japan

^g^Collaboration Research Center for Advanced Energy Materials, National Research and Innovation Agency, Institut Teknologi Bandung, Bandung, 40132, Indonesia

^h^Tsukuba Institute for Advanced Research (TIAR), University of Tsukuba, 1-1-1 Tennodai, Tsukuba, Ibaraki, 305-8577, Japan

***Corresponding Author**

Yoshikazu Ito; Email: [ito.yoshikazu.ga@u.tsukuba.ac.jp](mailto:ito.yoshikazu.ga@u.tsukuba.ac.jp)

Tatsuhiko Ohto; Email: [ohto@nagoya-u.jp](mailto:ohto@nagoya-u.jp)

**^†^Equal contribution**

**Supplementary discussion**

Our research target is the concentrations of crossover molecules in the counter chamber and the target concentrations of X-molecule were roughly estimated by the crossover flux quantity using the report [S1].

The crossover current density ($j_{crossover}$) was estimated by

$$j_{crossover}=M_{fuel}N_{e}F$$

where *M*_fuel_ is the molar flux of fuel crossover (based on a CO_2_ detector signal), *N*_e_ is the number of electrons per molecule of fuel that are transferred in the fuel cell's preferred electrochemical reaction, and *F* is the Faraday constant. For formic acid and methanol, the values of *N*_e_ are 2 and 6, respectively. Thus, the *M*_fuel_ can be estimated when we determine the $j_{crossover}$ with gas chromatography method for detecting CO_2_ emitted from the fuel cell. Assuming that the volume containing crossovered molecules at the cathode side (i.e. an intentionally overestimated 0.1 cm thickness water layer from Nafion membrane) is 0.1 mL per MEA area (cm^2^), the estimated crossover molar concentrations of fuel molecules between the Nafion membrane (cathode side) and carbon paper (facing to the Nafion) exceed 0.5 M (see the table). Thus, we set the 0.5 M concentration for initial understanding of crossover characters of X-molecules in our manuscript.

| Crossover current | $M_{fuel}$ (mmol/cm^2^/h) | Fuel concentration (mol/L) after 1 hour operation near the Nafion membrane |
| --- | --- | --- |
| 10 mA/cm^2^ formic acid | 0.187 | 1.87 M |
| 10 mA/cm^2^ methanol | 0.062 | 0.62 M |
| 10 mA/cm^2^ formic acid | 1.87 | 18.7 M |
| 10 mA/cm^2^ methanol | 0.62 | 6.2 M |

**Supplementary Figures**


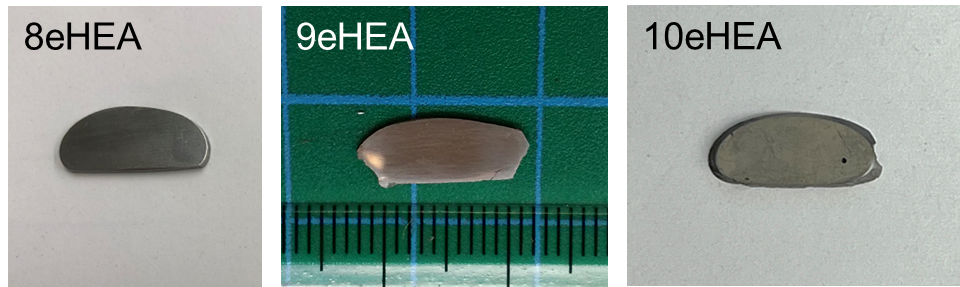


Figure S1. A photograph of a 9eHEA sheet.


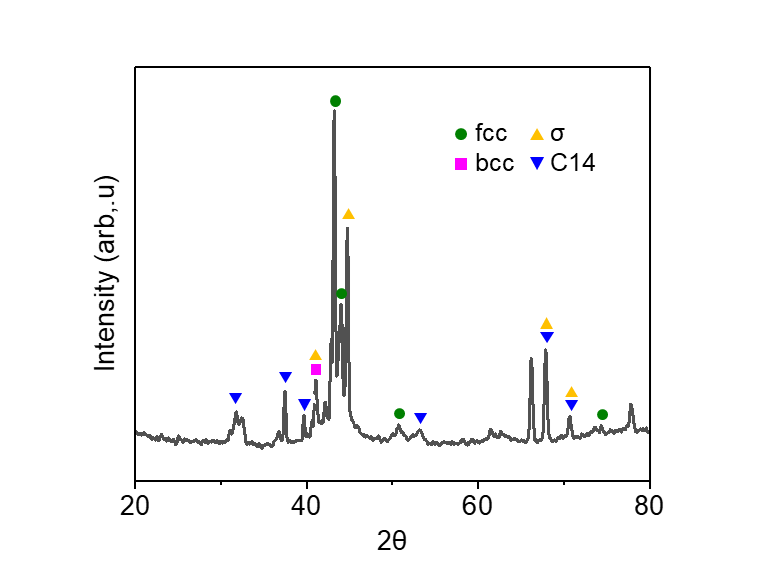


Figure S2. XRD spectra of 9eHEA sheet.

**
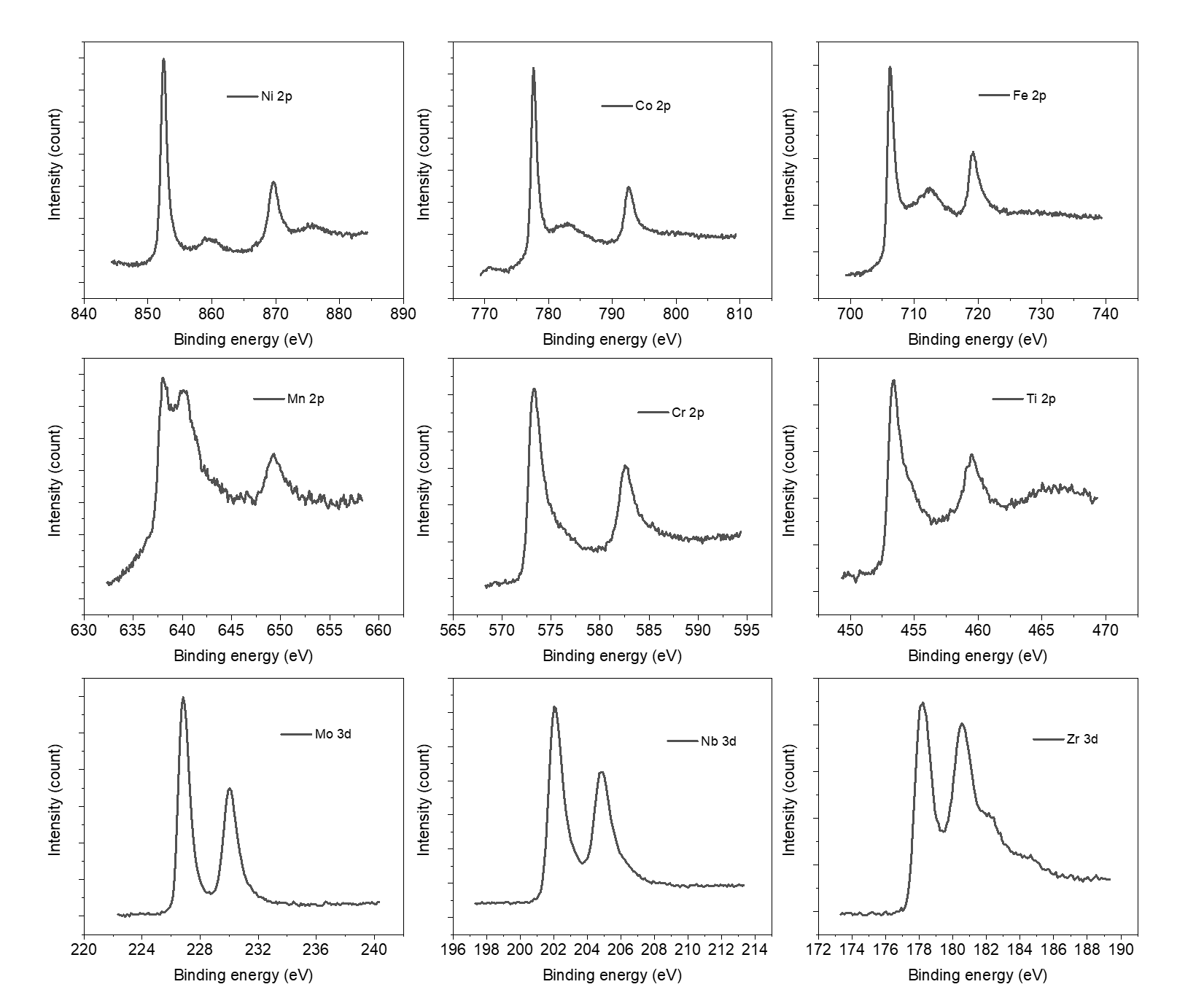
**

Figure S3. XPS spectra of as-prepared 9eHEA sheet. Some of metals were oxidized under air.


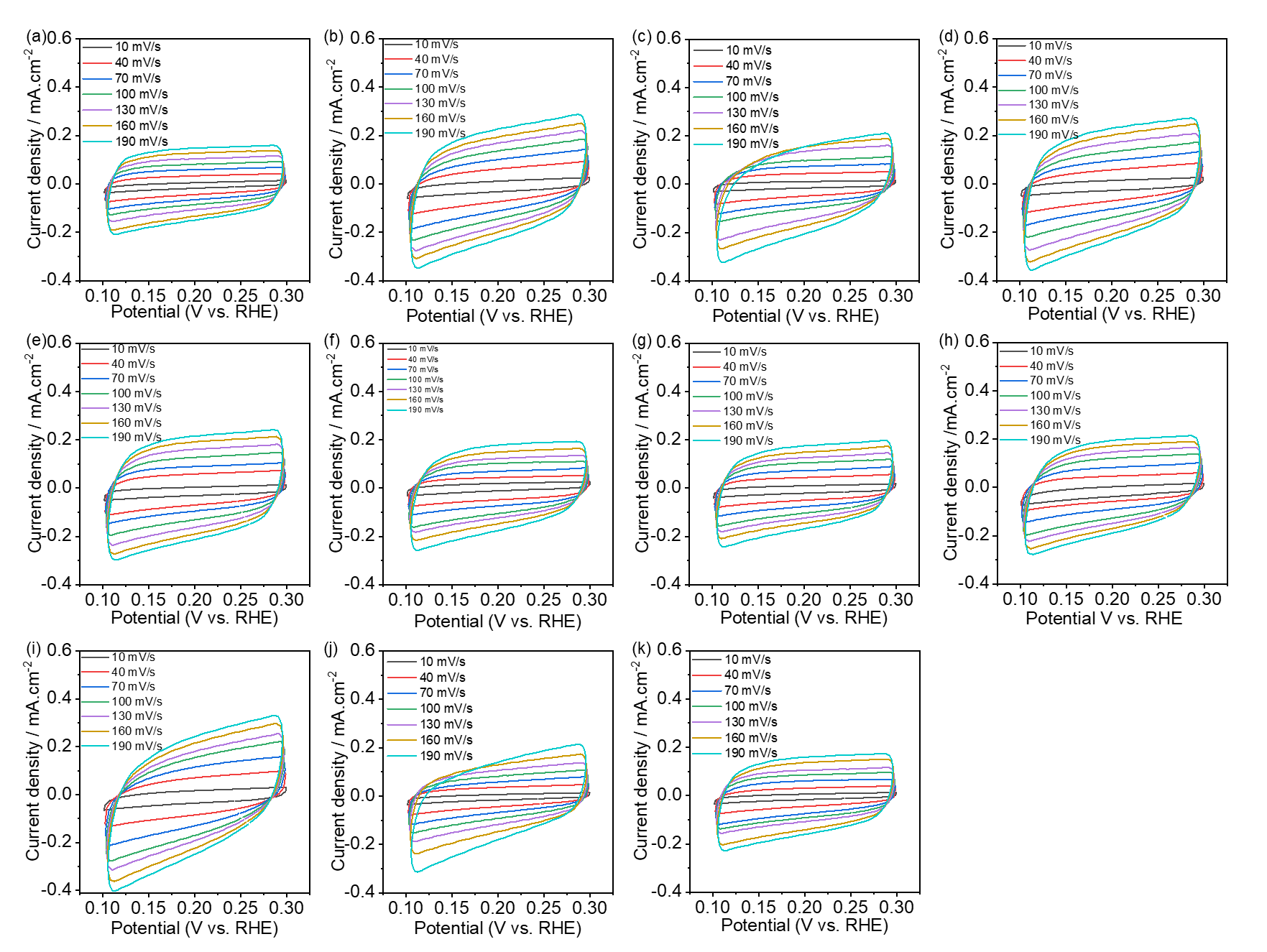

Figure S4. Cyclic voltammogram of 9eHEA anode in non-faradaic region of X-molecules such as (a) acetaldehyde, (b) biurea, (c) ethanol, (d) ethylene glycol, (e) formaldehyde, (f) formic acid, (g) glycerol, (h) no X-molecules, (i) lactic acid, (j) methanol and (k) urea with (l) the plot.

Figure S5. Electrochemical impedance spectroscopy of 9eHEA anode in 0.5 M H_2_SO_4_ electrolyte containing X-molecules or no X-molecule.

Figure S6. 1 h CA measurements of 9eHEA anode in 0.5 M H_2_SO_4_ electrolyte containing X-molecules for FT-IR measurements. The current density trends in Figure S6 were not matched with those in Figure 2(a) due to the use of different 9eHEA batch samples (the sample in Figure 2(a) was broken during FT-IR setup so that the different batch samples were employed for Figure S6).

Figure S7. FT-IR results on the surface of 9eHEA anode tested in 0.5 M H_2_SO_4_ electrolyte containing X-molecules from 1 h CA measurements.

Figure S8. XPS spectra of 9eHEA sheet after the CA test with (a-b) glycerol (C: 48.4 at.%, O: 29.1 at.% in total) and (c-e) urea (C: 32.3 at.%, O: 39.0 at.%, N: 10.0 at.% in total). The 9eHEA elements were not clearly detected due to the polymer influences.


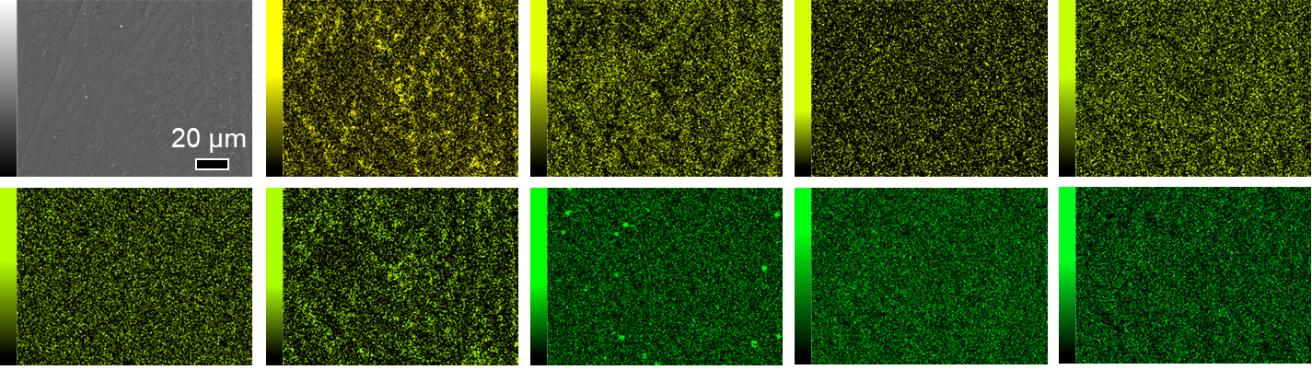


Figure S9. SEM-EDS mapping of the surface of 9eHEA anode tested in 0.5 M H_2_SO_4_ electrolyte without X-molecule from 1 h CA measurements.


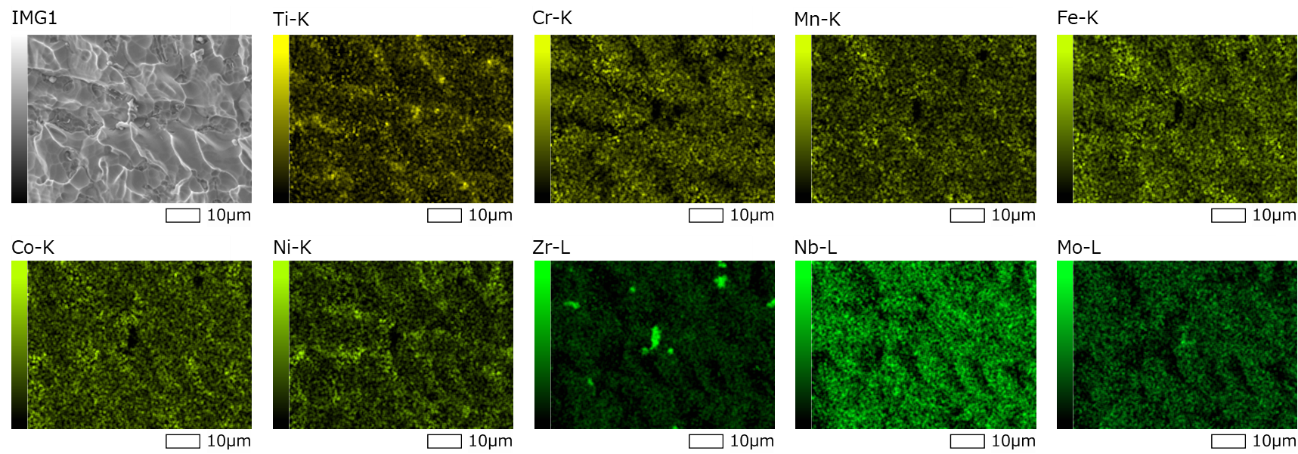


Figure S10. SEM-EDS mapping of the surface of 9eHEA anode tested in 0.5 M H_2_SO_4_ electrolyte containing acetaldehyde from 1 h CA measurements.


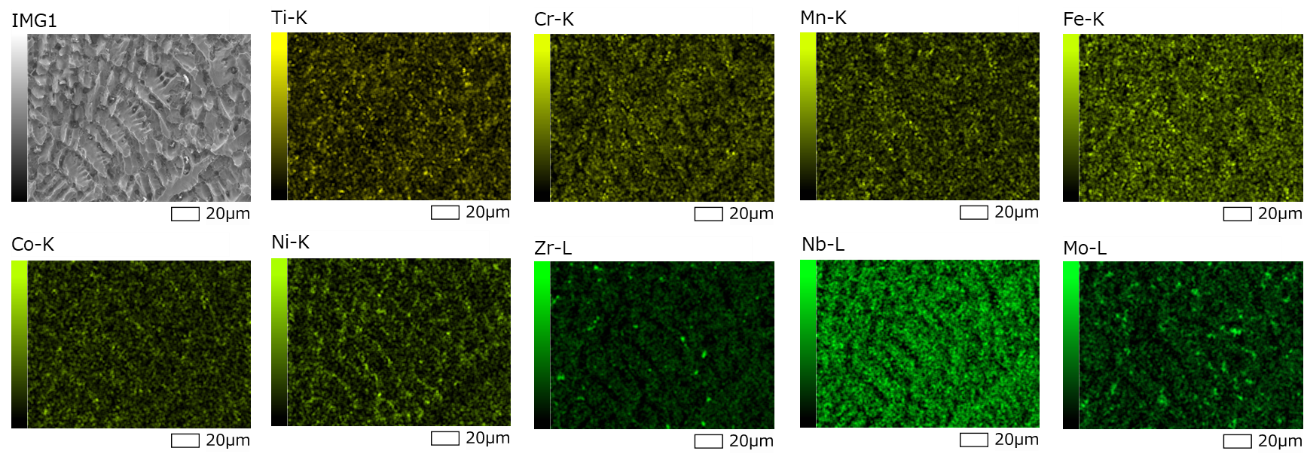


Figure S11. SEM-EDS mapping of the surface of 9eHEA anode tested in 0.5 M H_2_SO_4_ electrolyte containing biurea from 1 h CA measurements.


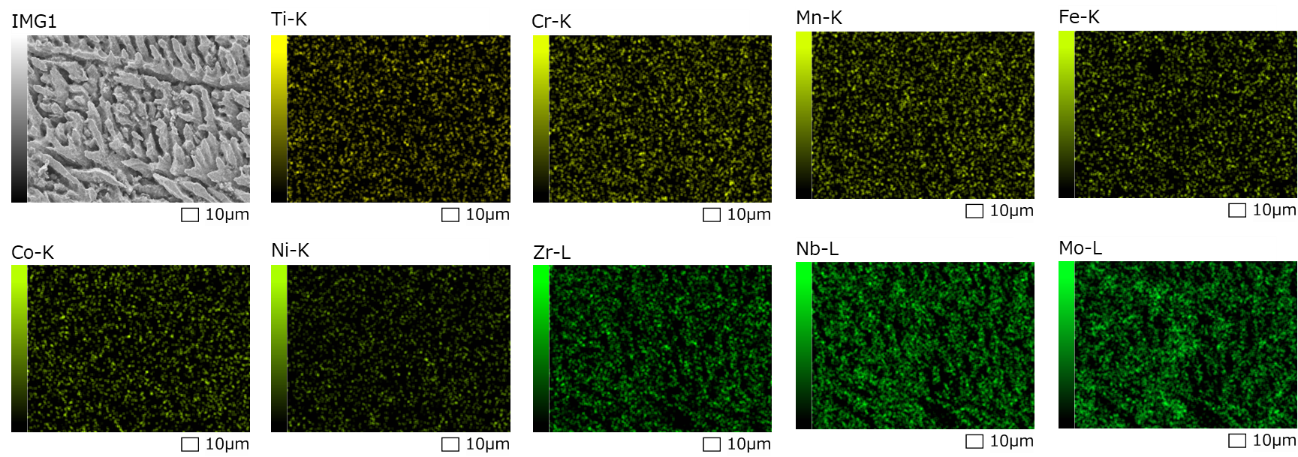


Figure S12. SEM-EDS mapping of the surface of 9eHEA anode tested in 0.5 M H_2_SO_4_ electrolyte containing ethylene glycol from 1 h CA measurements.


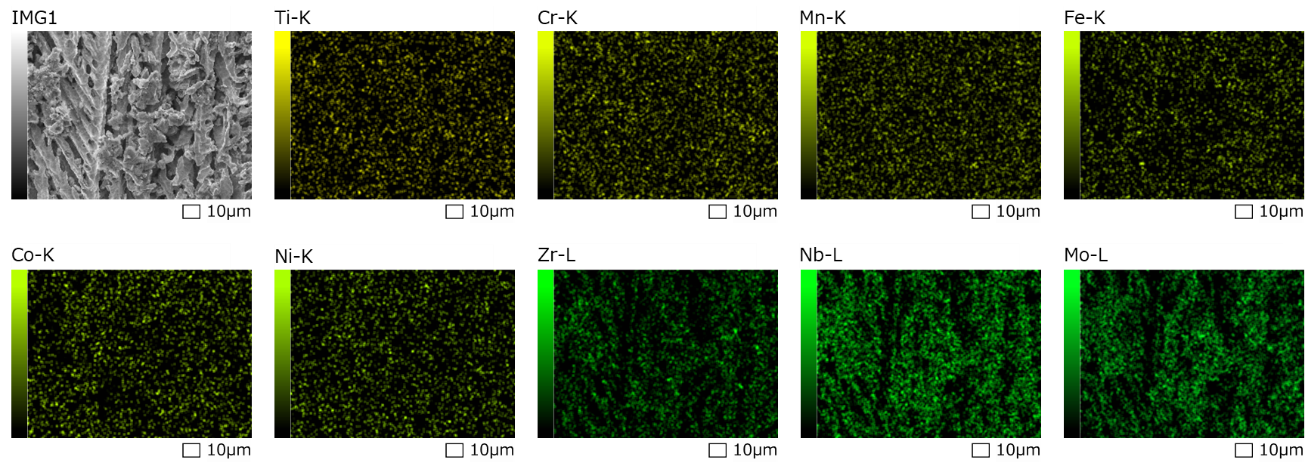


Figure S13. SEM-EDS mapping of the surface of 9eHEA anode tested in 0.5 M H_2_SO_4_ electrolyte containing ethanol from 1 h CA measurements.


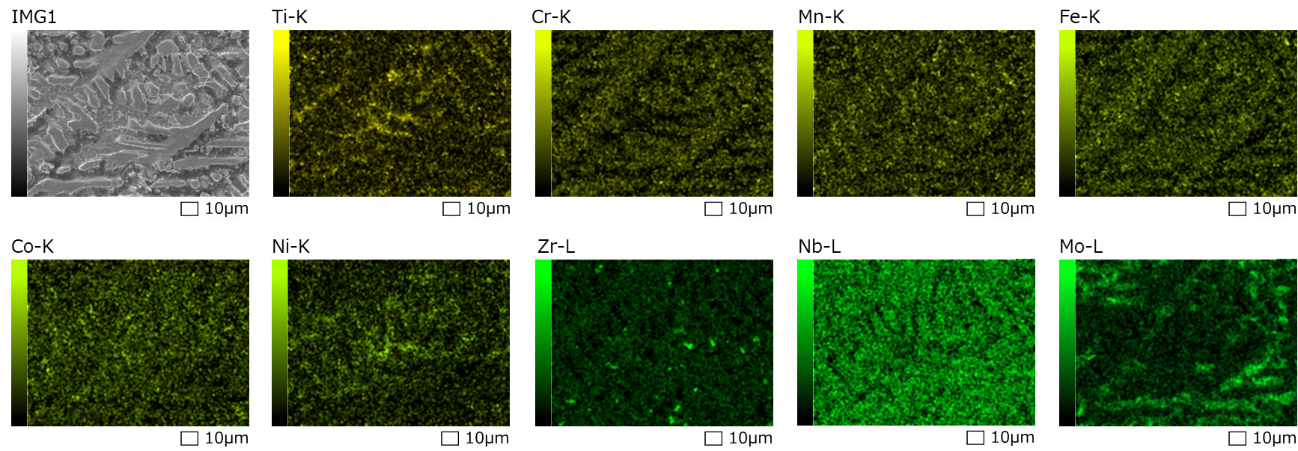


Figure S14. SEM-EDS mapping of the surface of 9eHEA anode tested in 0.5 M H_2_SO_4_ electrolyte containing formaldehyde from 1 h CA measurements.


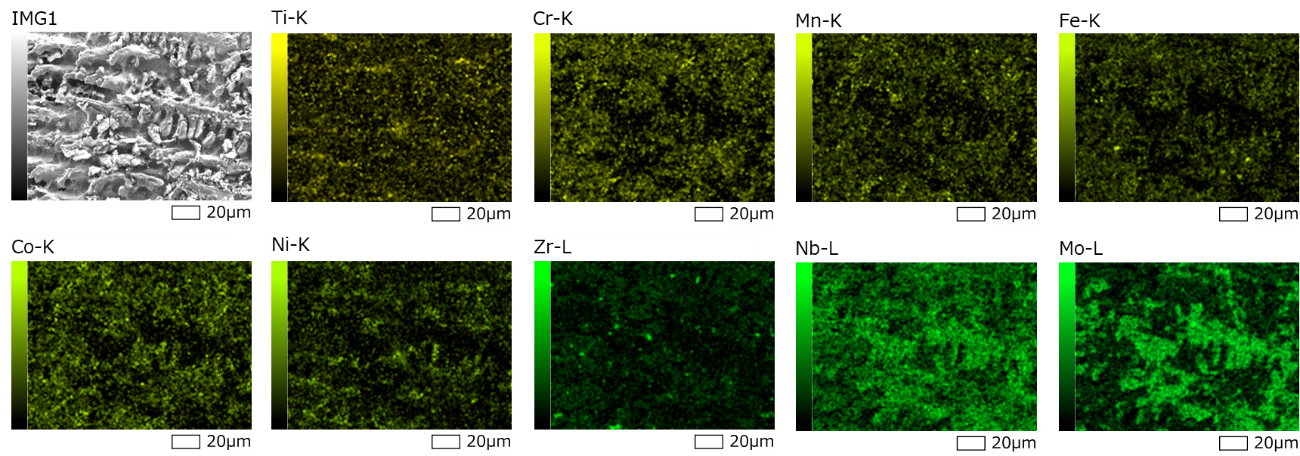


Figure S15. SEM-EDS mapping of the surface of 9eHEA anode tested in 0.5 M H_2_SO_4_ electrolyte containing formic acid from 1 h CA measurements.


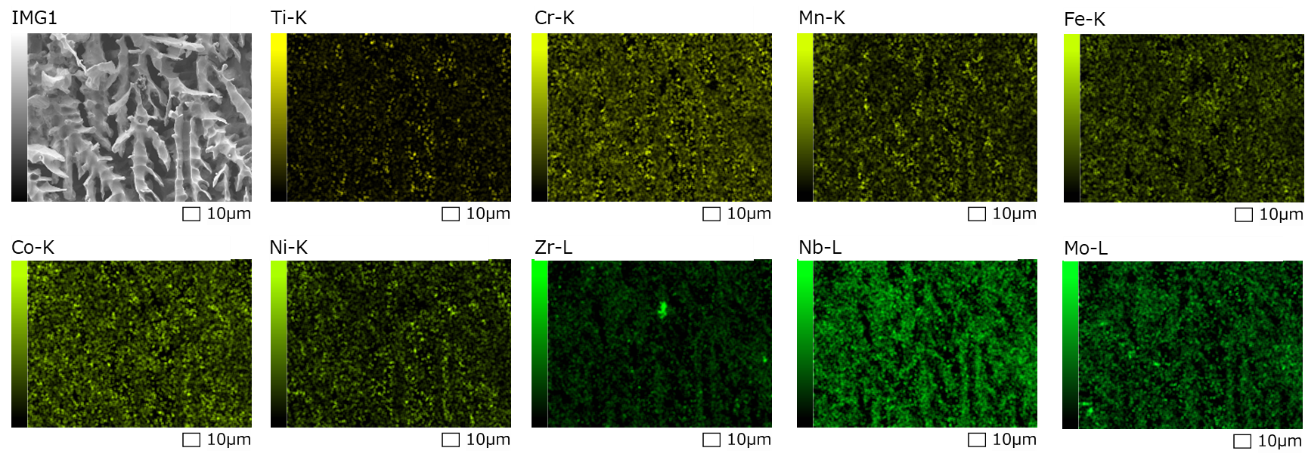


Figure S16. SEM-EDS mapping of the surface of 9eHEA anode tested in 0.5 M H_2_SO_4_ electrolyte containing glycerol from 1 h CA measurements.


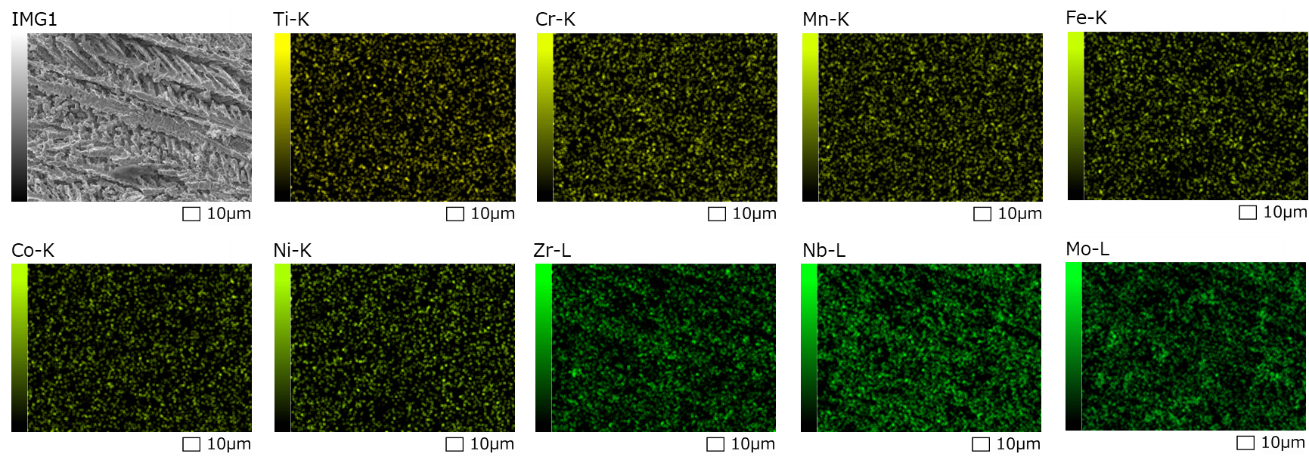


Figure S17. SEM-EDS mapping of the surface of 9eHEA anode tested in 0.5 M H_2_SO_4_ electrolyte containing methanol from 1 h CA measurements.


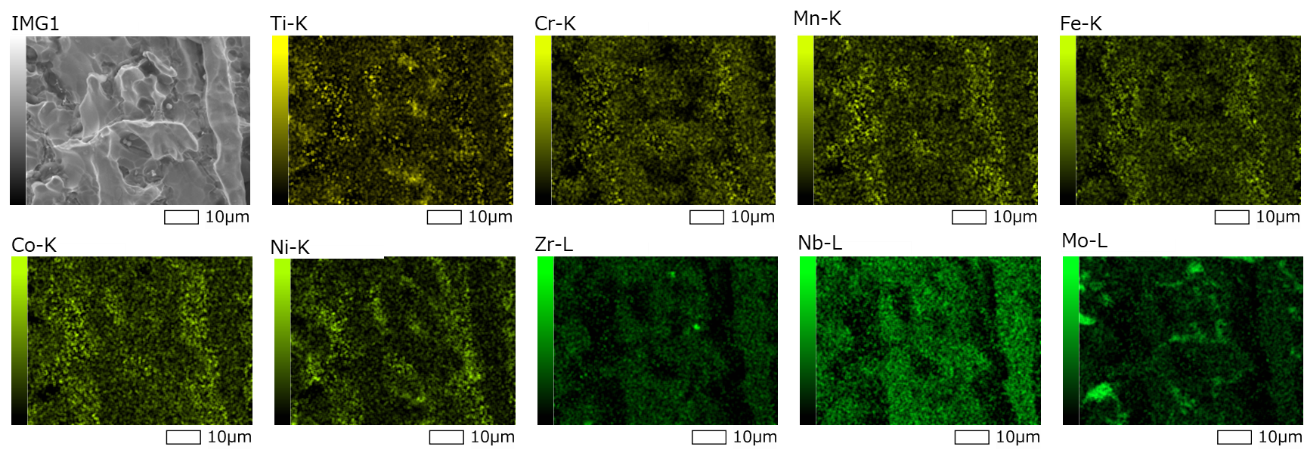


Figure S18. SEM-EDS mapping of the surface of 9eHEA anode tested in 0.5 M H_2_SO_4_ electrolyte containing lactic acid from 1 h CA measurements.


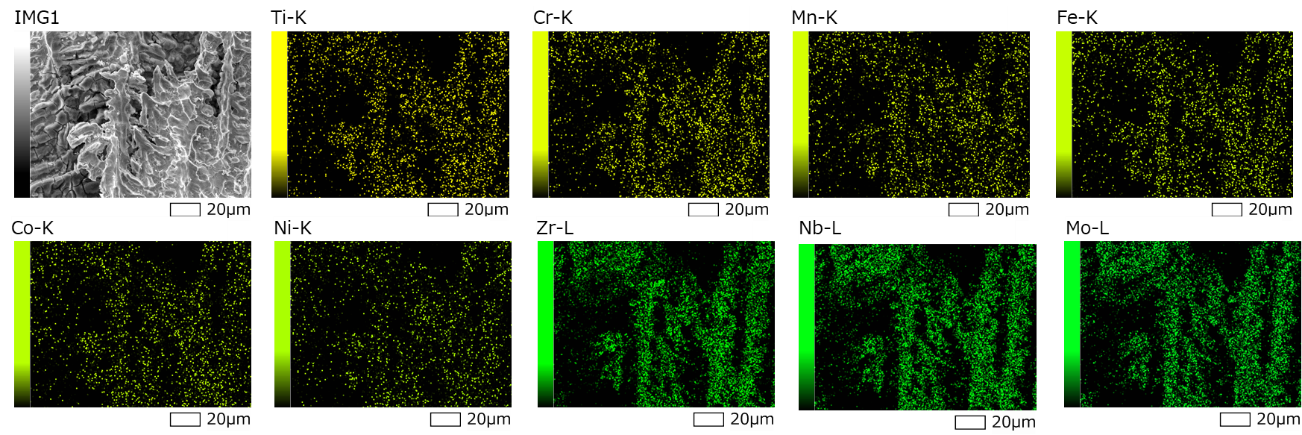


Figure S19. SEM-EDS mapping of the surface of 9eHEA anode tested in 0.5 M H_2_SO_4_ electrolyte containing urea from 1 h CA measurements.


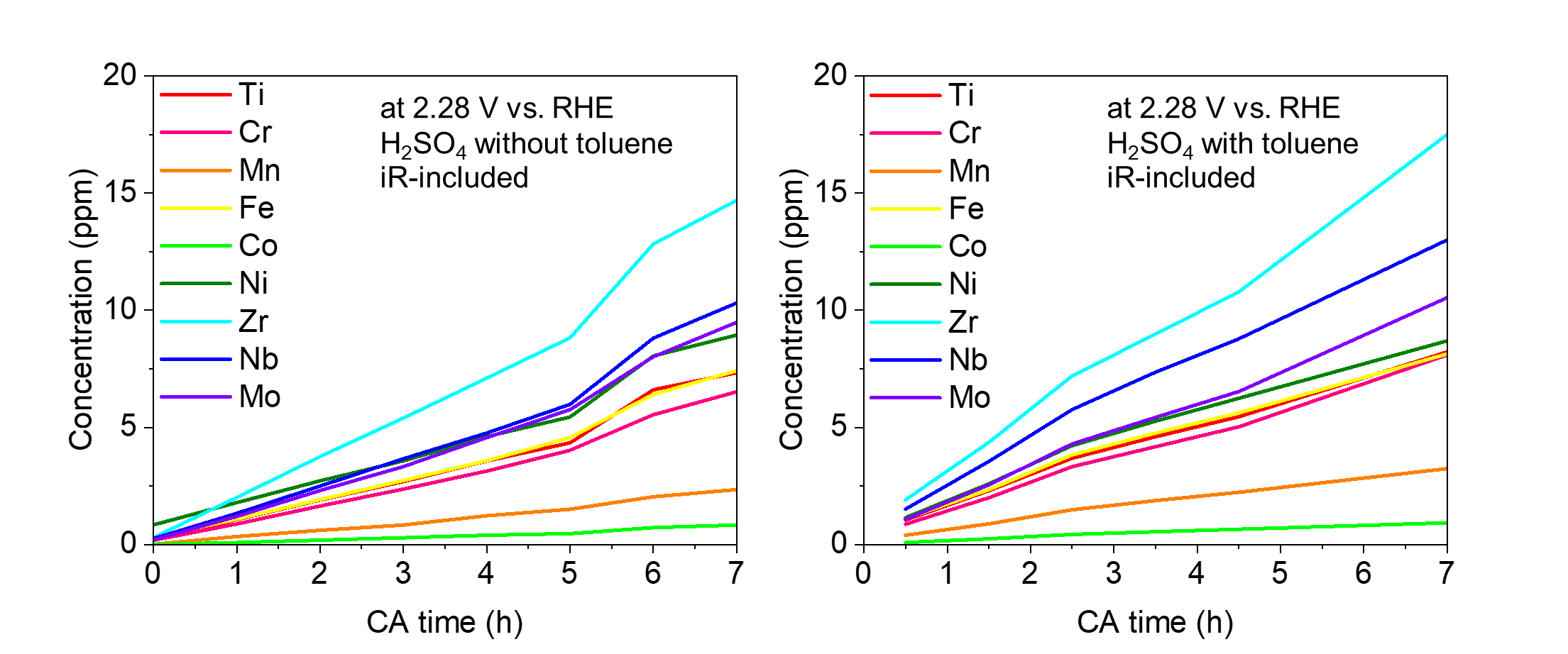


Figure S20. ICP-OES analysis of electrolyte in anode from the CA test with and without toluene. The leaching amount increased by 5-15% in the existence of toluene [S2].


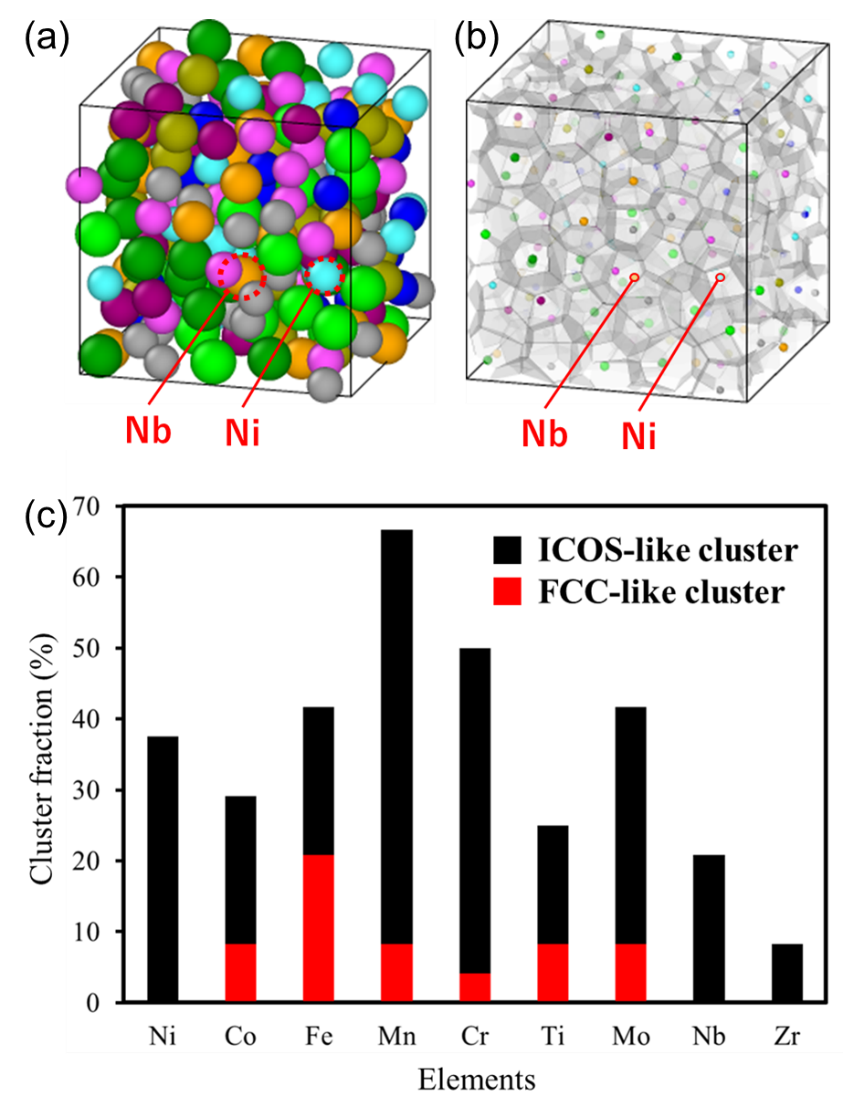


Figure S21. Voronoi tessellation. (a) Bulk structures of 9eHEA just before cutting the surface. Ni and Nb considered as active sites were indicated. (b) Voronoi tessellation of (a). (c) Fraction of the clusters for each atom. To investigate the local atomic environments of the active sites, we additionally performed the Voronoi tessellation for the bulk system just before cutting the surface [S3]. The bulk system in (a) was one of the structures annealed from 3000 K with the aid of machine-learning force field as previously reported [S4], whose simulated annealing processes can simulate as many phases appeared in the real crystal as possible. A Voronoi polyhedron is defined by Schläfli’s notation, 〈*n*_3_, *n*_4_, *n*_5_, *n*_6_〉, where *n*_i_ is the number of *i*-edged faces of a polyhedron. The Voronoi indices of standard BCC, FCC, and icosahedral (ICOS) are 〈0, 6, 0, 8〉, 〈0, 12, 0, 0〉, and 〈0, 0, 12, 0〉, respectively [S3]. The cluster consists of FCC and ICOS, while BCC (not shown here) also exists in other six bulk structures in our calculations. The target Ni and Nb were mainly involved in ICOS. This indicates that the active sites exist in other phases except BCC and FCC, or the interface between phases and glass structure.


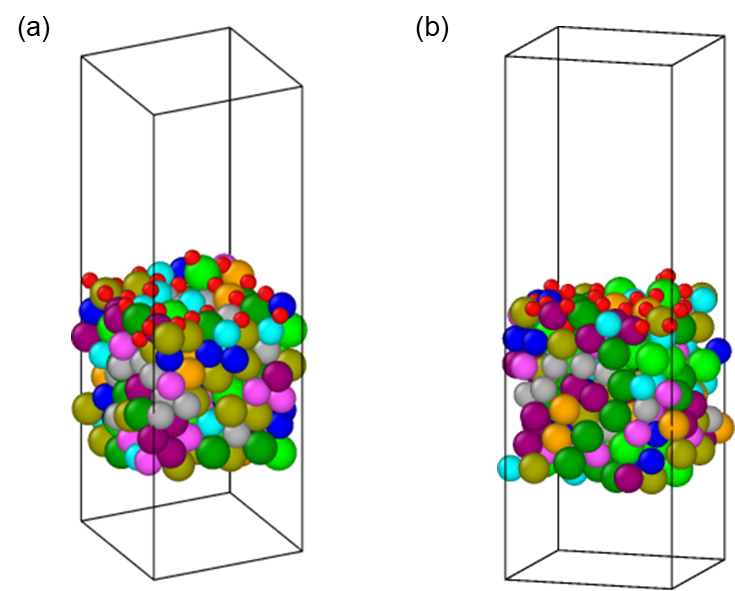


Figure S22. DFT calculation models of oxidized 9eHEA surface. (a) Ni-centered and (b) Nb-cantered structural outline. Both models are same.


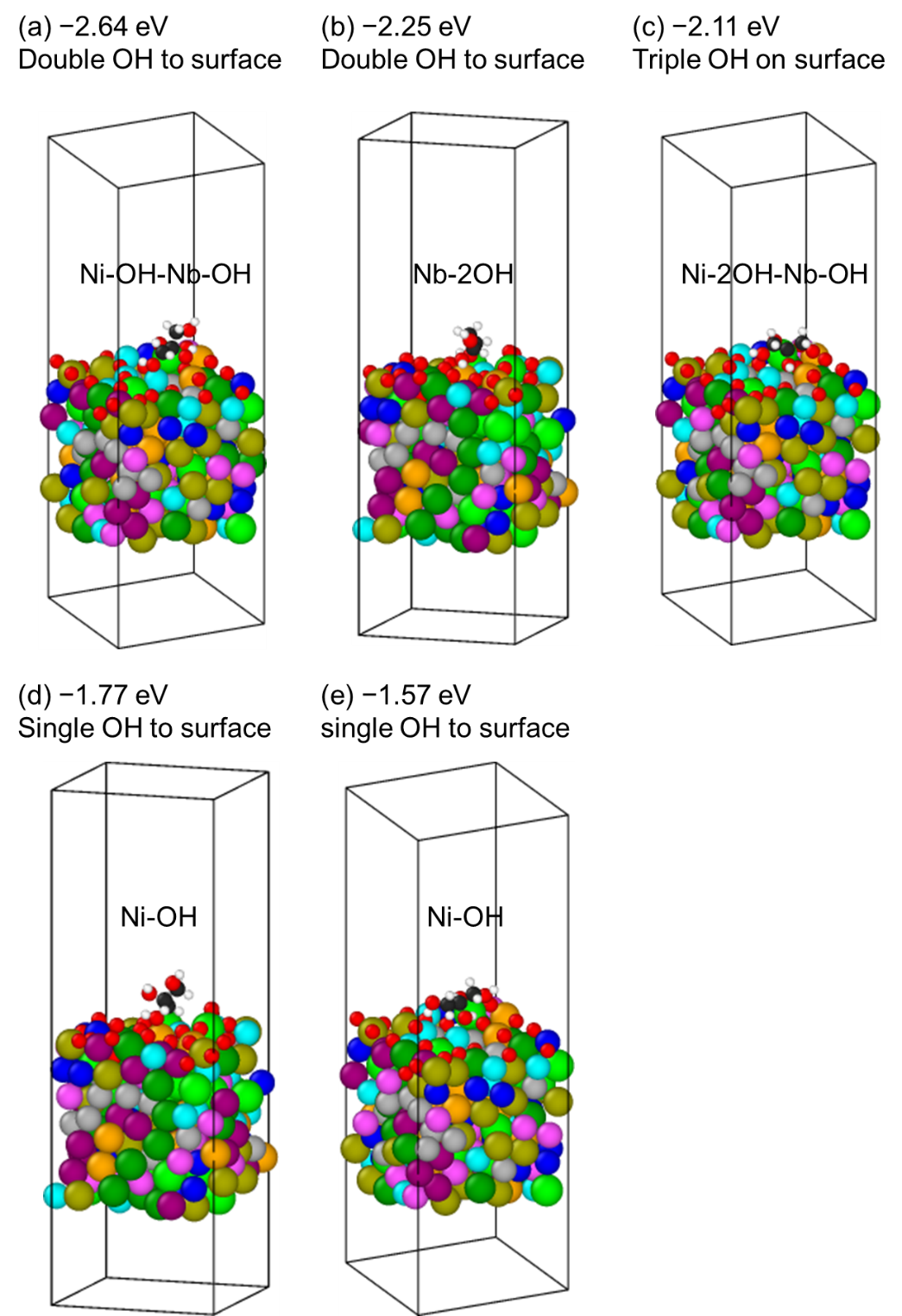


Figure S23. DFT calculations of glycerol adsorption (a) on Ni with single −OH (edge) and Nb with single −OH (center) as a bridge, (b) on Nb with double −OH (edge, center), (c) on Ni with double −OH and Nb with single −OH as a bridge, (d) on Nb with double −OH (edge, center), (e) on Ni with single −OH (edge) and (f) on Ni with single −OH (center) on the oxidized 9eHEA surface.


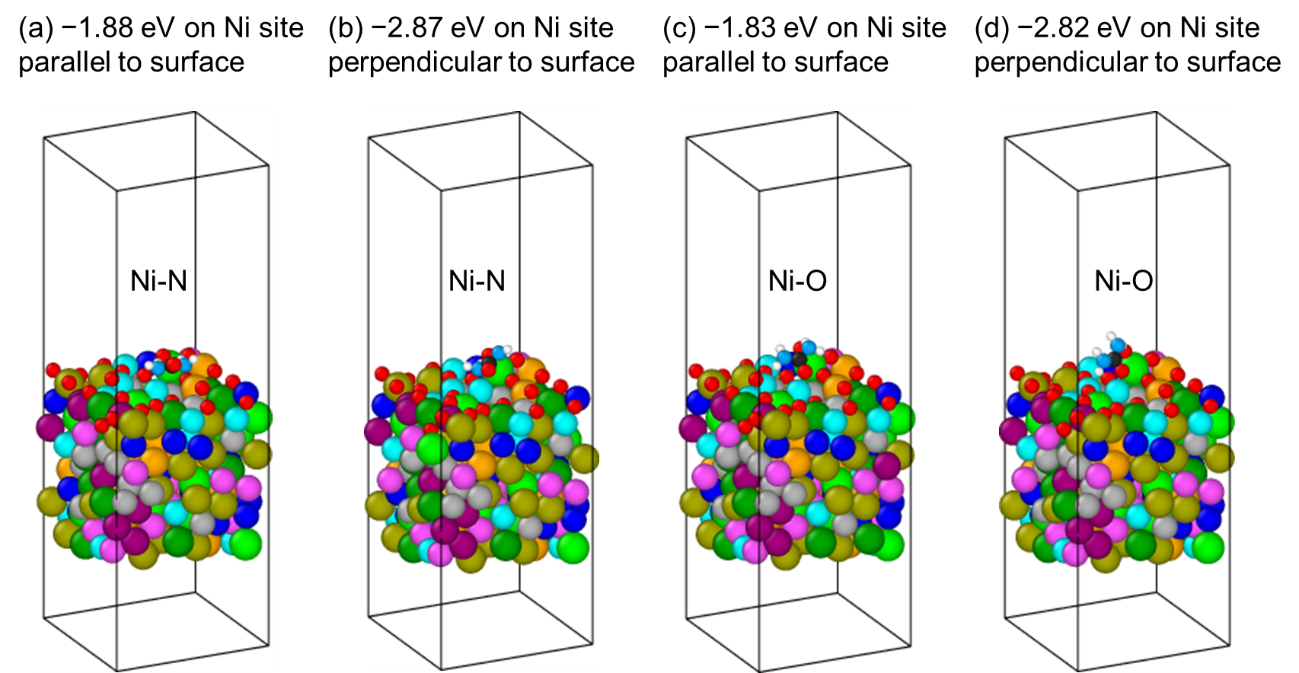


Figure S24. DFT calculations of urea adsorption (a) on Ni with −NH_2_ at a parallel position, (b) on Ni with −NH_2_ at a perpendicular position, (c) on Ni with C=O at a parallel position and (d) on Ni with C=O at a perpendicular position on the oxidized 9eHEA surface.


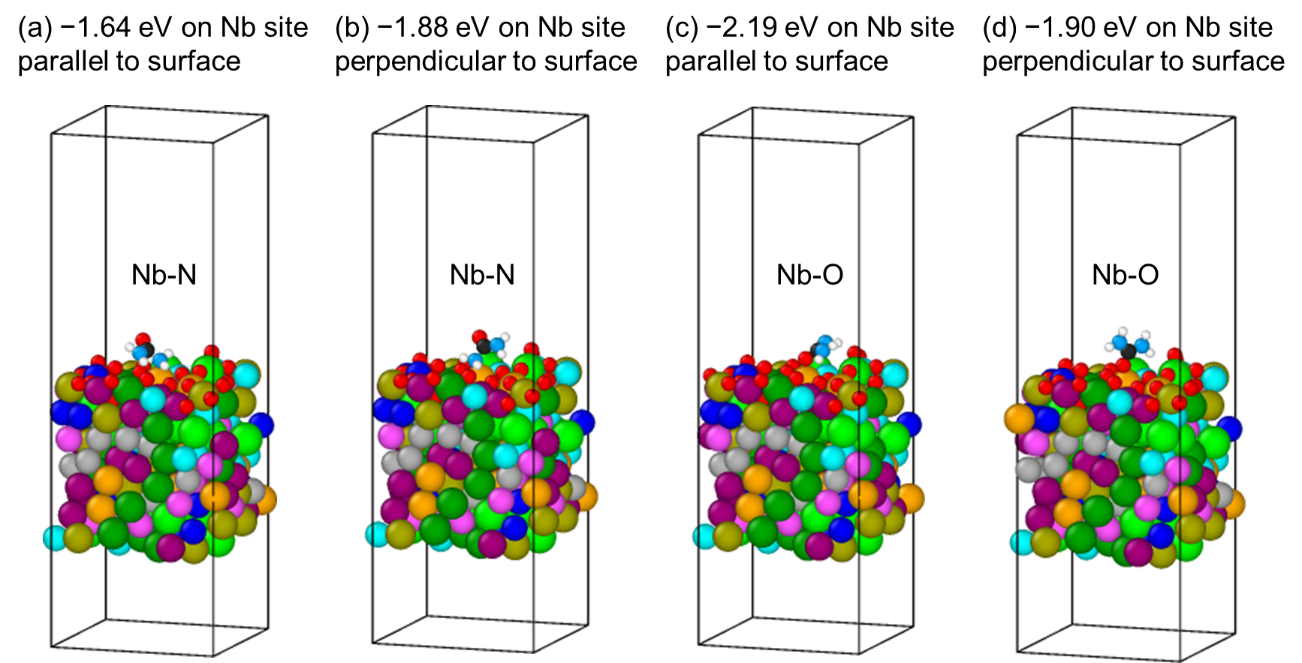


Figure S25. DFT calculations of urea adsorption (a) on Nb with −NH_2_ at a parallel position, (b) on Nb with −NH_2_ at a perpendicular position, (c) on Nb with C=O at a parallel position and (d) on Nb with C=O at a perpendicular position on the oxidized 9eHEA surface.

Table S1. Summary of electrochemical performance of 9eHEA anode tested in 0.5 M H_2_SO_4_ electrolyte containing X-molecules and no X-molecule.

|  | η_10_  (V) | η_100_  (V) | Tafel slope  (mV/dec) | C_dl_  (mF/cm^2^) | R_ct_  (Ohm) |
| --- | --- | --- | --- | --- | --- |
| No molecule | 0.72 | 0.86 | 126 | 22.0 | 861 |
| Methanol | 0.70 | 0.85 | 125 | 22.4 | 590 |
| Ethanol | 0.71 | 0.87 | 126 | 26.4 | 620 |
| Ethylene glycol | 0.74 | 0.97 | 122 | 34.7 | 555 |
| Glycerol | 0.68 | 0.83 | 122 | 26.1 | 450 |
| Formaldehyde | 0.70 | 0.84 | 134 | 32.0 | 458 |
| Acetaldehyde | 0.70 | 0.86 | 133 | 21.2 | 760 |
| Formic acid | 0.71 | 0.87 | 134 | 24.9 | 590 |
| Lactic acid | 0.71 | 0.84 | 130 | 37.5 | 640 |
| Urea | 0.72 | - | 175 | 22.4 | 928 |
| Biurea | 0.68 | 0.85 | 156 | 33.8 | 780 |

Table S2. Summary of atomic concentrations of 9eHEA anode before and after the test with and without X-molecules.

| 9eHEA with X-molecules after the CA test | Atomic concentration (at.%) | | | | | | | | |
| --- | --- | --- | --- | --- | --- | --- | --- | --- | --- |
|  | Ti | Cr | Mn | Fe | Co | Ni | Zr | Nb | Mo |
| acetaldehyde | 10.6 | 12.8 | 11.0 | 12.8 | 11.8 | 11.1 | 10.7 | 11.5 | 7.9 |
| biurea | 9.9 | 13.2 | 10.8 | 12.7 | 11.5 | 10.2 | 10.2 | 11.7 | 9.9 |
| ethylene glycol | 7.8 | 12.0 | 6.5 | 11.6 | 9.6 | 7.4 | 9.8 | 13.3 | 22.0 |
| ethanol | 8.5 | 14.0 | 5.3 | 12.7 | 10.7 | 6.9 | 9.0 | 13.9 | 18.9 |
| formaldehyde | 11.0 | 11.5 | 9.2 | 10.5 | 9.0 | 8.0 | 11.9 | 14.1 | 14.7 |
| formic acid | 8.3 | 10.9 | 8.8 | 10.4 | 8.8 | 7.2 | 8.3 | 19.5 | 17.7 |
| glycerol | 8.2 | 14.9 | 12.5 | 13.7 | 10.5 | 7.4 | 9.6 | 12.5 | 10.8 |
| methanol | 9.2 | 13.7 | 3.2 | 13.1 | 10.6 | 8.1 | 9.8 | 13.5 | 19.0 |
| lactic acid | 8.4 | 13.8 | 10.5 | 12.7 | 10.6 | 8.5 | 9.7 | 12.2 | 13.5 |
| urea | 9.9 | 8.6 | 7.8 | 8.9 | 8.9 | 9.0 | 9.5 | 22.1 | 15.3 |
| 9eHEA without X-molecules | Atomic concentration (at.%) | | | | | | | | |
|  | Ti | Cr | Mn | Fe | Co | Ni | Zr | Nb | Mo |
| as-synthesized | 10.8 | 11.0 | 8.3 | 11.6 | 12.3 | 11.8 | 10.9 | 11.4 | 11.9 |
| after test | 11.9 | 8.9 | 3.9 | 7.44 | 7.94 | 6.78 | 11.0 | 21.1 | 21.0 |

**References**

[S1] Jeong KJ, Miesse CM, Choi JH, et al. Fuel crossover in direct formic acid fuel cells, Journal of Power Sources. 2027;168:119–125. doi: 10.1016/j.jpowsour.2007.02.062

[S2] Tajuddin AAH, Ohto T, Tanimoto H, et al. Toluene-Poisoning-Resistant High-Entropy Non-Noble Metal Anode for Direct One-Step Hydrogenation of Toluene to Methylcyclohexane. ChemSusChem. 2025;18(2):e202401071. doi: 10.1002/cssc.202401071

[S3] Zhou W, Song J, Lin L, et al, npj Computational Materials, 2025;11:69. doi: 10.1038/s41524-025-01561-1

[S4] Tajuddin AAH, Wakisaka M, Ohto T, et al. Corrosion-Resistant and High-Entropic Non-Noble-Metal Electrodes for Oxygen Evolution in Acidic Media. Advanced Materials. 2023;35(3):2207466. doi: 10.1002/adma.202207466
